# BMP6 participates in the molecular mechanisms involved in APAP hepatotoxicity

**DOI:** 10.1007/s00204-024-03954-5

**Published:** 2025-01-19

**Authors:** Patricia Marañón, Stephania C. Isaza, Esther Rey, Patricia Rada, Yaiza García-García, James W. Dear, Carmelo García-Monzón, Ángela M. Valverde, Javier Egea, Águeda González-Rodríguez

**Affiliations:** 1 Unidad de Investigación, Hospital Universitario Santa Cristina, Instituto de Investigación Sanitaria Princesa (IIS-P), Madrid, Spain; 2 Instituto de Investigaciones Biomédicas Sols-Morreale (IIBM), Consejo Superior de Investigaciones Científicas-Universidad Autónoma de Madrid, Madrid, Spain; 3 Centro de Investigación Biomédica en Red de Diabetes y Enfermedades Metabólicas Asociadas (CIBERDEM), Madrid, Spain; 4 Centre for Cardiovascular Science, Queen’s Medical Research Institute, University of Edinburgh, Scotland, UK; 5 Centro de Investigación Biomédica en Red de Enfermedades Hepáticas y Digestivas (CIBEREHD), Madrid, Spain

**Keywords:** Bone morphogenetic protein, BMP6, APAP, ALF, Biomarker

## Abstract

Given the lack of accurate diagnostic methods of acetaminophen (APAP)-induced acute liver failure (ALF), the search for new biomarkers for its diagnosis is an urgent need. The aim of this study was to evaluate the role of bone morphogenetic protein 6 (BMP6) in APAP-induced ALF progression and its potential value as a biomarker of ALF. Hepatic and circulating BMP6 expression was assessed in APAP-treated mice and in serum samples from patients with APAP overdose. In addition, BMP6 expression and release was evaluated in hepatocytes after APAP exposure. BMP6 gene was silenced in Huh7 cells prior to APAP treatment and the culture medium (CM) was added to THP1 cells to evaluate the paracrine effects of hepatocyte BMP6 on APAP toxicity. Hepatic and serum BMP6 levels were increased in mice after APAP-induced ALF. In addition, a positive correlation was observed between circulating BMP6 and ALT activity in patients exposed to APAP overdose. Moreover, hepatocytes expressed and released BMP6 to the CM after APAP treatment. Indeed, the CM from APAP-treated Huh7 cells upregulated M1 and M2 markers in THP1 monocytes. The CM from BMP6-silenced Huh7, which was depleted of BMP6, reduced the expression of M2 markers in THP1 cells. In fact, expression of M2 markers was increased in THP1 cells exposed to BMP6. This study reveals that hepatic BMP6 expression is increased in APAP-induced acute liver injury, positioning it as a potential new biomarker of liver damage severity. Moreover, our data indicate that BMP6 might play a role in the hepatocyte–macrophage crosstalk during APAP-induced hepatotoxicity.

**Supplementary Information:**

The online version contains supplementary material available at 10.1007/s00204-024-03954-5.

## Introduction

Acute liver failure (ALF) is defined as the development of hepatocellular dysfunction in patients without previous hepatic disease in a period between 7 days and 26 weeks (Whitehouse and Wendon 2013). The key event for ALF development is the massive increase of hepatocyte death that overloads the regenerative capacity of the liver (Chung et al. 2012; Cardoso et al. 2017). ALF can be triggered by autoimmune diseases, viral infections or drug-induced liver injury (DILI) (Whitehouse and Wendon 2013; Lee et al. 2008). Acetaminophen (APAP) overdose is the main cause of DILI particularly in western countries (Cardoso et al. 2017; Bernal and Wendon 2013).

Even though APAP is highly safe at a therapeutic dose (Du et al. 2016), this commonly used analgesic and antipyretic drug can become hepatotoxic after an intentional or unintentional overdose along with alcohol consumption, fasting or combination with some herbal supplements, leading to glutathione depletion and accumulation of N-acetyl-p-benzoquinone imine (NAPQI), a highly reactive species derived from APAP metabolism in the liver (Bunchorntavakul and Reddy 2018; Yan et al. 2018). Excess of NAPQI forms protein adducts with cellular proteins, especially mitochondrial proteins, inducing electron chain dysfunction and eventually massive hepatocyte cell death that cannot be counteracted by the regenerative capacity of the liver (Ramachandran and Jaeschke 2018; Du et al. 2016). In addition, hepatocyte massive death by necrosis leads to the release of damage-associated molecular patterns (DAMPs) (Chung et al. 2012) that bind to toll-like receptors (TLRs), thereby promoting an inflammatory response through the activation of the liver resident macrophages (Kupffer cells) and the subsequent release of cytokines and chemokines (Ramachandran and Jaeschke 2018). These mediators activate other cells from the immune system, such as neutrophils and monocytes, which are recruited to the damaged area to remove cellular debris and prepare the tissue for repair (Rada et al. 2018).

Since APAP-induced ALF is characterized by a fulminant progression that can lead to end-stage liver disease or liver transplantation, an early intervention is essential and therefore, an accurate diagnosis and prognosis are of utmost importance. For this reason, great efforts are being made in searching new serological biomarkers for prognosis. In this regard, the mitochondrial protein glutamate dehydrogenase (GLDH) and mtDNA have been proposed as markers for prognosis of DILI; however, poor sensitivity and poor predictive values of liver injury and mortality were reported for both (McGill and Jaeschke 2018). Likewise, microRNA-122 has also been proposed as a specific biomarker of prognosis in APAP-induced DILI (McGill and Jaeschke 2018; Andrade et al. 2019), together with cytokeratin 18 (K18), both total and the cleaved product (ccK18), which has shown good accuracy in predicting early injury in APAP-overdose patients, and outcome in patients with DILI (McGill and Jaeschke 2018; Umbaugh and Jaeschke 2021; Llewellyn et al. 2021). Currently, APAP–protein adducts are the most promising markers for diagnosis, especially APAP–cysteine adducts (APAP-CYS) whose detection is to date the only biomarker proposed for diagnosis of DILI (Heard et al. 2011; Vliegenthart et al. 2015).

Bone morphogenetic proteins (BMPs) are soluble proteins belonging to the TGFβ superfamily. More than 20 BMPs have been identified and classified into subfamilies based on their structural and functional features. Even though these proteins were first discovered for their capacity to induce bone formation, latter studies have associated BMPs with the development and homeostasis of different organs and systems (Wozney et al. 1988; Carreira et al. 2014; Xiao et al. 2007). Regarding their implication in ALF, elevations in BMP2 and BMP4 in the liver were found in animal models of ALF and their signaling cascades have been implicated in the process of liver repair (Nakatsuka et al. 2007; Oumi et al. 2012).

Moreover, BMP6 has been widely studied in the liver due to its key role in iron homeostasis. It has been found that BMPs modulate the expression of hepcidin which, in turn, regulates hepatic and circulating iron concentration (Silvestri et al. 2019). While in basal conditions, regulation of hepcidin gene *HAMP* is mediated by BMP2, in situation of iron overload, hepatic BMP6 expression is increased and its signaling pathway leads to increased hepcidin expression in an attempt to reduce iron concentration (Andriopoulos et al. 2009; Canali et al. 2017; Meynard et al. 2009). In fact, different studies have evidenced the implication of BMP6 in preclinical models and patients with iron overload (Kautz et al. 2011; Rausa et al. 2015; Daher et al. 2016). In this regard, iron overload may be an ALF trigger and, in fact, two studies have established a relationship between serum hepcidin concentration and the prognosis of ALF (Simonse et al. 2013; Spivak et al. 2019).

Based on this background, the aim of this study was to elucidate whether BMP6, which has already been shown to be involved in the regulation of iron homeostasis in the liver, could play a role in ALF and be clinically useful as a marker of presence of liver damage.

## Materials and methods

### Animals

Animal experimentation was carried out following both Spanish and European legislations. Animals were housed in controlled conditions of temperature (22 °C) and humidity with dark/light cycles of 12 h, at the Instituto de Investigaciones Biomédicas Sols-Morreale (Madrid, Spain) animal facilities (PROEX 007/2019). Mice were fed with standard chow diet ad libitum and had free access to drinking water.

Male C57BL/6 J mice between 2 and 3 months of age were submitted to a model of APAP-induced ALF. Briefly, 30 mg of APAP (A7085 Merck Life Science, Darmstadt, Germany) was dissolved in 1.5 ml saline and warmed at 55 °C until totally dissolved. Overnight fasted mice were injected intraperitoneally with a single dose of 300 mg/kg APAP or saline (control group), and sacrificed 6 and 24 h after injection. Livers and serum samples were collected and conveniently stored for further analysis.

### Histopathology assessment

Livers collected from mice were embedded in paraffin and 5 μm-thick liver tissue slices were made. Liver sections were stained with Hematoxylin and Eosin (H&E) and representative images were taken using an optical microscope Nikon Eclipse E400 (Nikon, Tokyo, Japan) equipped with a plan Apocromatic 4X, 10X and 20X objective (Nikon). Hepatic necrosis area was assessed in six different lobular areas, using ImageJ software (NIH, Bethesda, MD, USA).

### Transaminase activity analysis

Blood samples were collected from animals after sacrifice and serum was used in a 1:4 dilution. ALT and AST activities were evaluated using colorimetric kits (41282, Spinreact, Girona, Spain, and MAK055, Merck Life Science, Darmstadt, Germany, respectively).

### BMP6 detection by immunohistochemistry (IHQ)

Liver tissue slides were deparaffinized and rehydrated. Antigen retrieval was performed following HIER method by boiling the slides for 20 min in 10 mM sodium citrate pH 6. Sections were blocked prior to immunostaining with anti-BMP6 antibody 1:100 (ab155963, Abcam plc, Cambridge, UK) overnight. After incubation with the secondary antibody, DAB detection system (EnVision™ Flex Mini Kit, High pH (Link) Agilent, Santa Clara, CA, USA) was used for visualization according to the manufacturer’s instructions. For histological assessment, six representative images were taken per section using a Nikon Eclipse E400 optical microscope (Nikon) equipped with a plan Apocromatic 4X, 10X and 20X objective (Nikon). Intensity of BMP6 staining was quantified using ImageJ software (NIH) and reported as the average value in arbitrary units (a.u.).

### Study population

This study included serum samples kindly provided by Professor James W. Dear from Centre for Cardiovascular Science, Queen’s Medical Research Institute, University of Edinburgh (Scotland, UK). Ethical approval for this study was provided by London—South East Research Ethics Committee (18/LO/0894) (ClinicalTrials.gov identifier: NCT03497104). Patients presenting to Royal Infirmary of Edinburgh, UK (RIE) following APAP overdose, who met the inclusion criteria, were asked to provide informed consent to participate in the prospective. Inclusion criteria: age 16 years and over, hospital attendance with APAP overdose alone or as part of a mixed overdose and patient is able to give informed consent. Exclusion criteria: patient detained under the Mental Health Act, inability to provide informed consent, unreliable history of overdose and prisoners.

Clinical characteristics of the study population are shown in Table 1: 18 patients with APAP overdose, from which 9 developed DILI, with ALT in serum values over 100 U/L (ALT = 4571 ± 3089 U/L), and 9 patients that did not develop DILI, reflected in normal serum levels of ALT (ALT = 16 ± 6.63 U/L). The serum samples from these patients were used to quantify the circulating concentration of BMP6. Serum samples analysis was blinded and performed in a random order.

**Table 1:** Characteristics of the study population

	No DILI (*n*=9)	DILI (*n*=9)
Age (years)	23.33 ± 8.2	37.33 ± 13.34*
Gender (female/male, %)	77.78 / 22.22	55.56 / 44.44
Time from ingestion of APAP to hospital presentation (hours)	24.06 ± 58.23	18 ± 17.87
Received NAC (%)	100%	100%
ALP (U/L)	75.78 ± 19.67	98.56 ± 34.28
Creatinine (µmol/L)	63 ± 11.43	65.11 ± 17.08
Urea (mmol/L)	4.056 ± 1.57	4.22 ± 2.32
WBC (x10⁹/L)	9.09 ± 3.75	6.96 ± 2.78
Potassium (mmol/L)	3.87 ± 0.17	3.91 ± 0.76
INR	1.08	3.3***
ALT (U/L)	16 ± 6.63	4571 ± 3089****
Bilirubin (µmol/L)	8.11 ± 2.93	38.44 ± 37.86****
Haemoglobin (g/L)	140.4 ± 18.28	129.1 ± 19.52
MCV (fl)	87.22 ± 7.96	90.78 ± 9.91
Sodium (mmol/L)	140.2 ± 1.09	136.6 ± 3.09

Data are shown as mean ± SD or as number of cases (%)

*NAC* Acetylcysteine; *ALP* Alkaline phosphatase; *WBC* White blood cell count; *INR* International normalized ratio; *ALT* Alanine aminotransferase; *MVC* Mean corpuscular volume

*p<0.05, ***p<0.005 and ****p<0.0001, DILI vs. No DILI

### Quantitative analysis of serum BMP6 levels by ELISA

Serum BMP6 concentration was quantified by ELISA using the mouse BMP6 ELISA kit (CSB-E09279m) for mouse samples and the human BMP6 ELISA kit (CSB-E09277h) for human samples (Cusabio Technology LLC, Hubei, China), following the manufacturer´s instructions. Absorbance from samples was interpolated to a standard curve using a four-parameter logistic (4-PL) equation.

### Cell culture and treatments

Immortalized neonatal mouse hepatocyte cell line (Pardo et al. 2015) and human hepatoma cell line Huh7 (ATCC, Manassas, VA, USA) were cultured in Dulbecco´s modified Eagle medium (DMEM, Cytiva, Marlborough, MA, USA) with high glucose supplemented with 10% heat-inactivated fetal bovine serum (FBS), HEPES and antibiotics (penicillin/streptomycin). Human leukemia monocytic-derived cell line THP1 (kindly provided by Dr. Elena Fernández-Ruiz, Hospital Universitario de la Princesa, Madrid, Spain) was cultured in suspension in RPMI (RPMI 1640, Gibco) supplemented with 10% heat-inactivated FBS and antibiotics (penicillin/streptomycin). All cells were maintained at 37 °C, 5% CO_2_ and relative humidity 95%.

To simulate APAP-induced damage, immortalized mouse hepatocytes and Huh7 cells were stimulated with APAP dissolved in ethanol at different concentrations (1 mM, 5 mM, 10 mM and 20 mM), or ethanol (control) for various time periods. For treatment with BMP6, THP1 cell line was serum-starved for 3 h, and then 1, 10 or 100 ng/ml of recombinant human BMP6 protein (507-BP/CF, R&D Systems, Inc. Minneapolis, USA) was added to the culture media. After treatment, culture media were collected and plates were washed with PBS and used for further analysis.

### Transient silencing of BMP6 in Huh7 cells

After a confluence of 60% was achieved, Huh7 cells were transfected with small interfering RNA (siRNA) for BMP6 (siBMP6) or siRNA control (siControl, siC) (siGENOME SMARTpool Human BMP6 M021475-03–0005, siGENOME™ Control Pool Non-Targeting #1 D-001206-13-20, Dharmacon™ Inc., Lafayette, CO, USA) at a final concentration of 10 nM in serum-free DMEM without antibiotics for 36 h. Quantification of *BMP6* gene silencing was evaluated by quantitative real-time PCR (RT-qPCR) (Table S1) and western blot analysis.

### Cell survival analysis

Cell survival was measured with crystal violet staining. Plates were washed with PBS to remove unattached death cells and covered with 0.2% crystal violet diluted in 2% ethanol for 30 min. Then, staining excess was removed by washing with distilled water and plates were left to dry. Finally, colorant was dissolved in 1% sodium dodecyl sulfate (SDS) and optical density was measured with spectrophotometer Spectra MR (29,010, Dynex Technologies, Chantilly, VA, USA) at 560 nm. Cell viability was calculated from the absorbance measurement.

### Cytotoxicity assay by lactate dehydrogenase (LDH)

Cellular toxicity was evaluated by measuring lactate dehydrogenase (LDH) release to the culture media due to cellular necrosis. Assay was performed using the Cytotoxicity Detection KitPLUS LDH (0474492600, Roche Diagnostics, Mannheim, Germany) following manufacturing indications. Percentage of cytotoxicity was calculated as percentage of cytotoxicity = ((Sample O.D.–Control O.D.) / (Positive control O.D–Control O.D.)) × 100.

### DAPI staining

Immortalized mouse hepatocytes and Huh7 cells were grown on glass coverslips and treated with APAP as described previously in this section. After treatment, culture media were removed and cells were washed with PBS. Afterward, cells were permeabilized and fixed with methanol for 10 min. DAPI (4ʹ,6-diamidino-2-phenylindole) staining (Thermo Fisher Scientific Inc., Madrid, Spain) was added in 1:1000 proportion for 5 min after which excess was washed with PBS. Mounting medium used was Fluoromont G® (BioNova cientifica, Madrid, Spain). Representative images were taken using an optical microscope Nikon Eclipse E400 (Nikon) equipped with a plan Apocromatic 40X objective (Nikon). Presence of apoptotic nuclei was assessed with ImageJ software (NIH).

### ROS quantification in vitro by fluorescence microplate reader

Cells were seeded in a 96 multi-well plate in a density of 2 × 10^4^ cells/plate. The probe dihydroethidium (DHE, 2,140,299, Invitrogen by Thermo Fisher Scientific Inc.) was used at a concentration of 10 μM in DMEM FluoroBrite containing High Glucose and without L-Glutamine (Thermo Fisher Scientific Inc.) to measure superoxide. After 30 min with the probe, vehicle or APAP was added and ROS production was measured in a fluorescence microplate reader (CLARIOstarPlus BMG Labtech, Germany) for 2 h using 488/550–580 excitation/emission filter pairs. DAPI staining was used to normalize number of cells (358/455–465). Each condition was run in triplicate and antimycin A (10 μM) was used as a positive control.

### Gene expression analysis

Total RNA was extracted from liver tissue and cell lysate using TRIzol reagent (Vitro, Sevilla, Spain). Obtained RNA purity and concentration were measured with Nanodrop (Termofisher Nanodrop 2000c) and cDNA was obtained by reverse transcription of RNA using ImProm-II™ Reverse transcription kit (Promega Inc., Madison, WI, USA) in a T100TM Thermal Cycler (BioRad Inc., Madrid, Spain). RT-qPCR was performed with SYBR Green method using StepOnePlusTM Real-Time PCR System sequence detector (Thermo Fisher Scientific, Inc.) and quantified with ΔΔCt method. Samples were run in duplicate and normalized with endogenous gene *36b4*. Primer sequences used are listed in Table S1.

### Western blot analysis

Protein content from culture media was precipitated with trichloroacetic acid (TCA; 0.85 g/ml), washed with acetone (cooled at −20 ºC) and boiled in Laemmli buffer. Total protein extracts from cell lysates were obtained using RIPA buffer (50 mM Tris–HCl, pH 7.4, 1% Triton X-100, 0.2% SDS, 1 mM EDTA, 1 mM PMSF and 5 μg/ml leupeptin) and boiled in Laemmli buffer. Protein samples were separated in 10% or 15% SDS-PAGE by electrophoresis following with transferring to Immunoblot nitrocellulose membrane. After blocking with 3% BSA, membranes were incubated overnight with primary antibodies at 4 °C: anti-BMP6 (1:1000) (ab155963, Abcam plc), anti-JNK (sc-7345, Santa Cruz Biotechnology Inc., Heidelberg, Germany), anti-cleaved caspase-3 (#9661), anti-phospho-JNK (#4668), anti-phospho-P38 MAPK (#9211) and anti-P38 (#9212) (Cell Signalling Technology, Danvers, MA, USA). Finally, membranes were incubated with the corresponding secondary antibody (Santa Cruz Biotechnology Inc.). Immunoreactive bands were visualized using the ECL Western blotting protocol (Bio-Rad). Densitometric analysis of the bands was performed using Image J software (NIH). Anti-Tubulin (sc-166729, Santa Cruz Biotechnology Inc.), anti-vinculin (sc-73614, Santa Cruz Biotechnology Inc.) and Ponceau staining were used as loading control.

### Statistical analysis

Quantitative variables are expressed as measures of central tendency (mean) and dispersion (standard error of mean, SEM). Data between groups were compared with Student´s t test for variables following a normal distribution and Mann–Whitney U test for continuous variables following a non-parametric distribution. All statistical analyses were performed using GraphPad Prism 6.0 (GraphPad Software Inc., San Diego, CA, USA) and IBM SPSS Statistics 21.0 (SPSS Inc., IBM, Armonk, NY, USA) software, with a *p*-value of < 0.05 considered as statistically significant.

## Results

### Hepatic BMP6 expression is increased in an animal model of APAP-induced acute liver injury

**Fig. 1:**
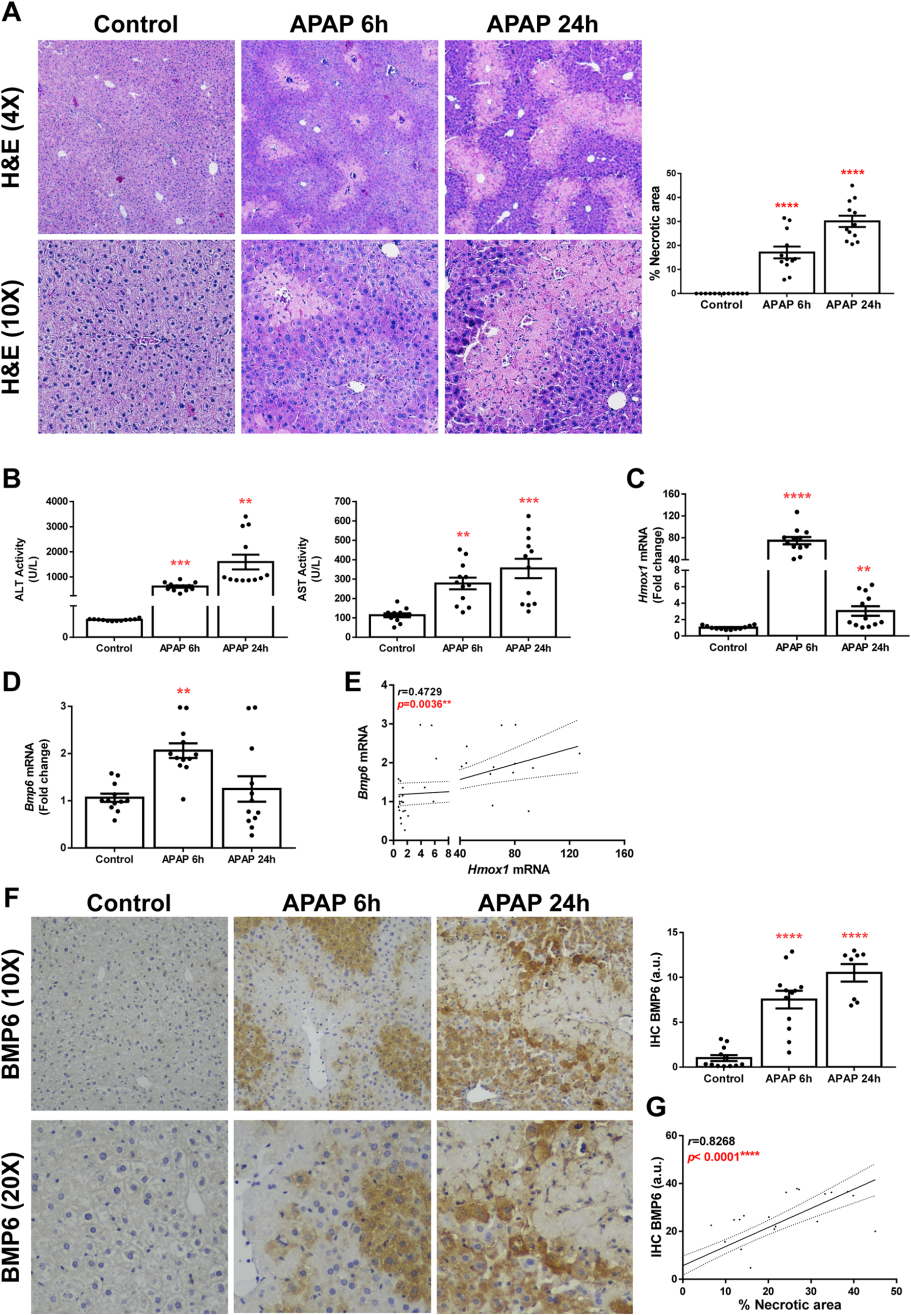
Hepatic BMP6 expression is increased in an animal model of APAP-induced ALF. **A** Representative 4X and 10X images of H&E and percentage of necrotic area quantification (%). **B** ALT and AST activity determination in serum samples. **C and D** *Hmox1* and *Bmp6* mRNA levels determined by RT-qPCR and normalized to *36b4* gene expression. Data are expressed as fold of change relative to control condition (Control). **E** Correlation of *Bmp6* mRNA expression with *Hmox1* mRNA expression. **F** Representative 10X and 20X images of BMP6 immunostaining from liver sections and its quantification. Data are expressed as arbitrary units (a.u.). **G** Correlation of BMP6 hepatic expression with percentage of necrotic area. Experimental conditions: Mice i.p. injected with vehicle (saline) or APAP (300 mg/kg) and sacrificed 6 or 24 h after APAP administration (*n* = 12 animals per group). **p < 0.01, ***p < 0.005 and ****p < 0.0001, APAP 6 h or 24 h *vs.* Control

In order to elucidate the role of BMP6 in acute liver injury, we conducted an experimental model of APAP-induced ALF. For this purpose, overnight fasted mice were injected intraperitoneally with a dose of 300 mg/kg of APAP and sacrificed after 6- or 24-h post-injection. As expected, necrotic areas were observed in the liver tissue of these animals (Fig. 1A), as well as an increase in ALT and AST activity in serum of mice treated with APAP compared to control animals receiving saline (Fig. 1B). The increase of both necrotic area and transaminases was more evident after 24 h post-APAP overdose. It is well-known that in a situation of APAP overdose, antioxidant mechanisms are activated in order to counteract oxidative stress subsequent to glutathione depletion (Reid et al. 2005). Notably, mRNA levels of heme-oxigenase 1 (*Hmox1*), a well-known regulator of antioxidant systems, were increased in mice challenged with APAP, particularly at 6 h after injection (Fig. 1C). Then, we measured the hepatic expression of BMP6 in liver samples and observed an increased expression 6 h after APAP overdose (Fig. 1D). In fact, hepatic mRNA levels of *Bmp6* significantly correlated with *Hmox1* mRNA levels (Fig. 1E). Likewise, augmented BMP6 protein content was detected by immunostaining of liver sections from APAP-injected animals compared to the controls (Fig. 1F) and a significant positive correlation was found between BMP6 protein expression and the percentage of necrotic area in the liver tissue (Fig. 1G).

### Circulating BMP6 is increased in mice with APAP-induced acute liver injury and in patients with APAP overdose

**Fig. 2:**
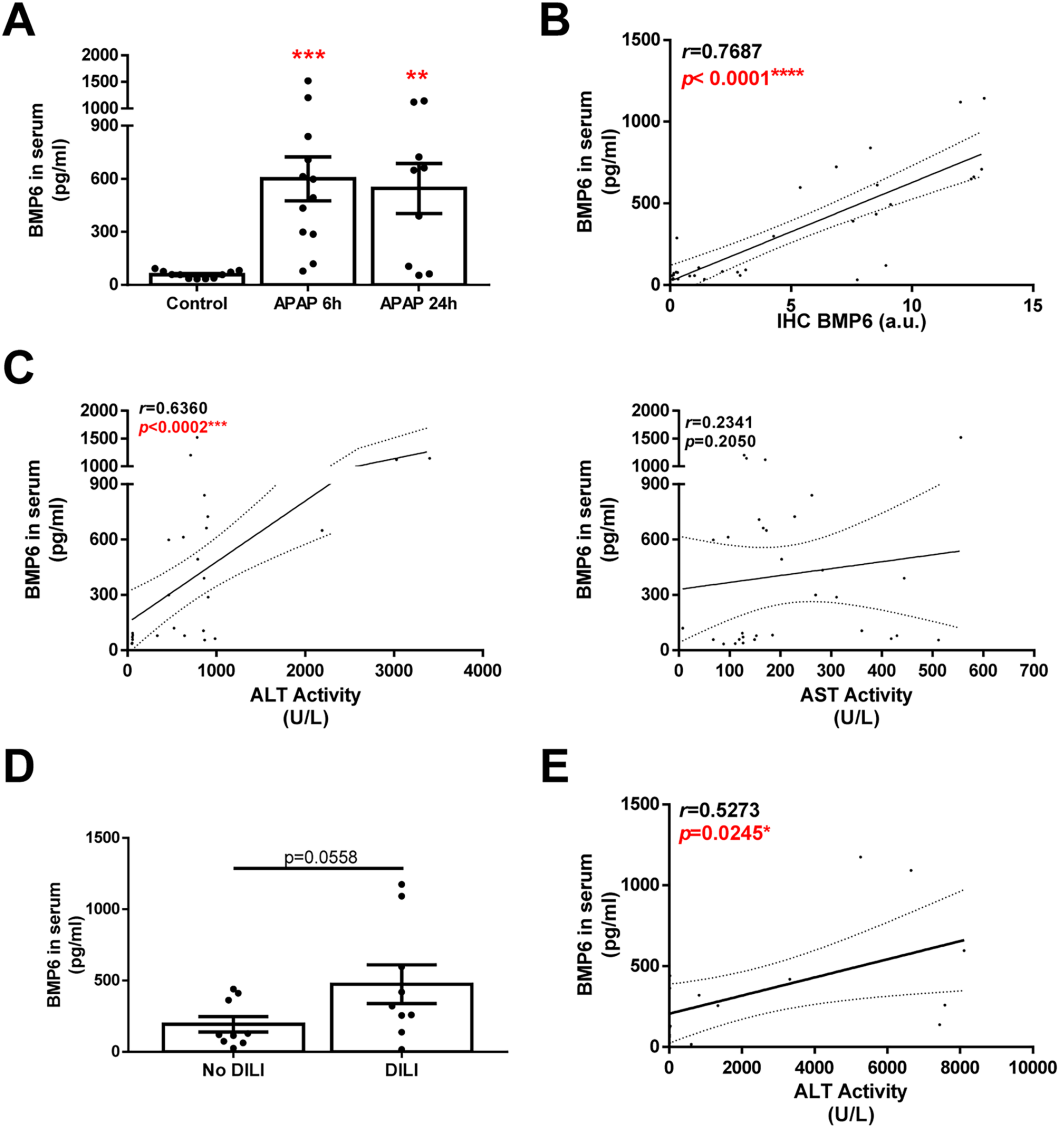
Circulating BMP6 is increased in mice after APAP overdose and in patients with APAP intoxication. Experimental conditions: Mice i.p. injected with vehicle (saline) or APAP (300 mg/kg) and sacrificed 6 or 24 h after APAP administration (*n* = 12 animals per group). **A** Serum levels of BMP6 determined by ELISA. Data are expressed as pg/ml. **B and C** Correlation of serum BMP6 levels with hepatic expression of BMP6, circulating ALT and AST, respectively. Study population: 18 patients with APAP overdose, 9 with DILI (ALT > 100 U/L) and 9 without DILI. **D** Serum levels of BMP6 determined by ELISA. Data are expressed as pg/ml. **E** Correlation of serum BMP6 levels with circulating ALT levels. **p < 0.01 and ***p < 0.005, APAP 6 h or 24 h *vs.* Control

Taking into account the previous results, we sought to explore whether the elevation of BMP6 in the liver contributes to increase BMP6 in serum. For this purpose, BMP6 was determined in serum samples from APAP-treated mice. As shown in Fig. 2A, increased serum BMP6 concentrations were found in APAP-treated mice. In addition, serum BMP6 levels positively correlated with hepatic BMP6 expression (Fig. 2B), as well as with serum ALT values (Fig. 2C).

Next, we determined BMP6 concentrations in serum samples from a cohort of 18 patients with APAP intoxication; 9 of them developed DILI reflected by ALT levels over 100 U/L. In fact, liver injury was severe since ALT levels in most of these patients were higher than 1000 U/L (mean ALT values, 4571 ± 3089 U/L). Circulating BMP6 levels were higher in patients with DILI (474.7 ± 407.7 pg/mL, *n* = 9) than in those that did not develop liver injury (193.8 ± 163.2 pg/mL, *p* = 0.0558, *n* = 9, Fig. 2D). In fact, circulating levels of BMP6 positively correlated with serum ALT values (*r* = 0.5273, *n* = 18, *p* = 0.0245, Fig. 2E).

### Immortalized mouse hepatocytes express and release BMP6 after APAP insult

**Fig. 3:**
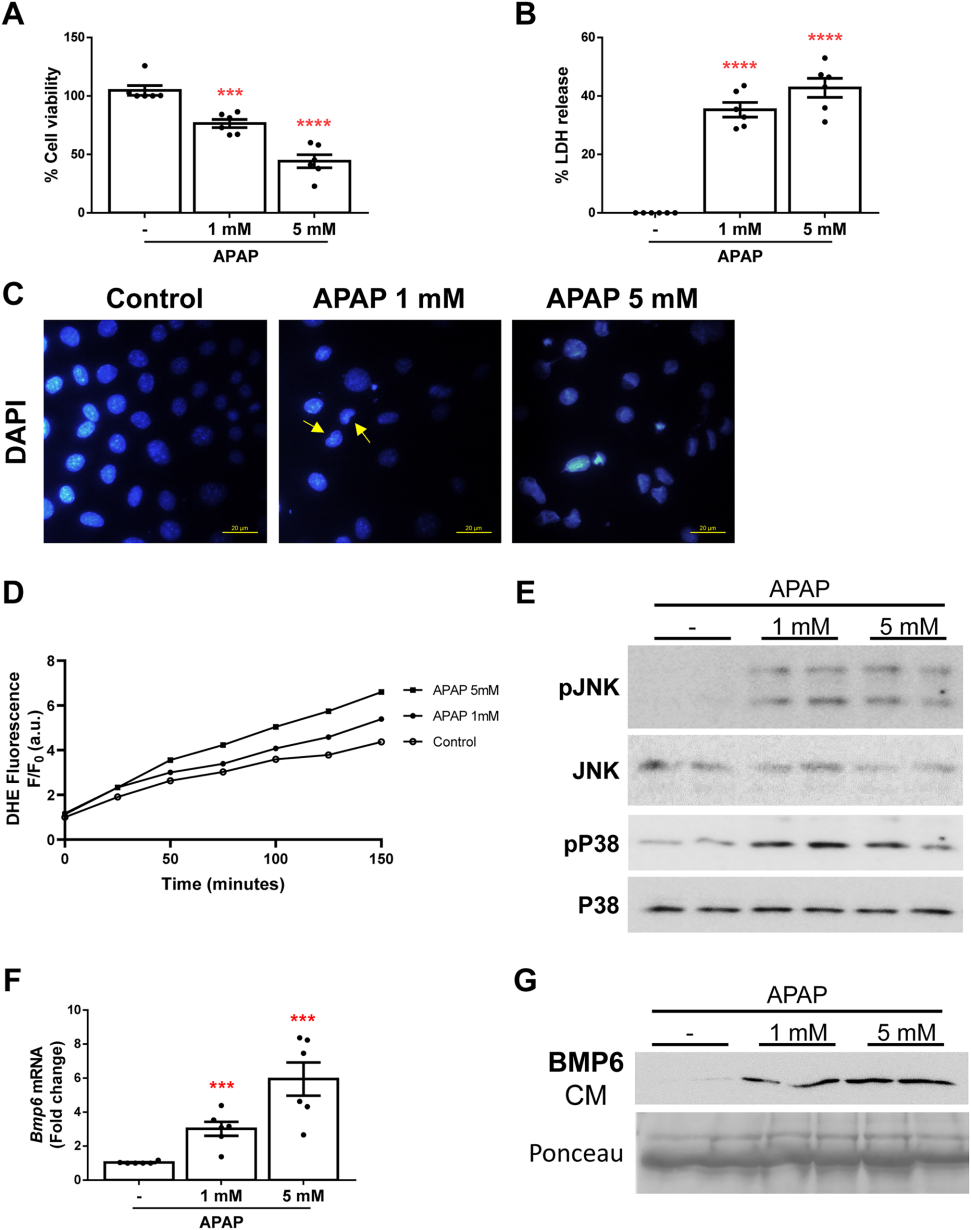
Immortalized mouse hepatocytes showed elevated expression and release of BMP6 after APAP treatment. **A** Cell viability determined by crystal violet staining. Data are expressed as percentage relative to control condition (100%). **B** Cytotoxicity determined by lactate dehydrogenase (LDH) release. Data are expressed as percentage relative to the positive control (100%). **C** Representative 40X images of DAPI staining. **D** ROS production detected with DHE probe represented as DHE fluorescence F/F_0_ (a.u.). **E** Representative blots of the cell lysates with the indicated antibodies. **F** *Bmp6* mRNA levels determined by RT-qPCR and normalized to *36b4* gene expression. Data are expressed as fold of change relative to control condition. **G** Representative blot of the cultured media (CM) with BMP6 antibody. Ponceau staining was used as loading control. Experimental conditions: immortalized mouse hepatocytes treated with 1 mM or 5 mM APAP for 2 (D), 6 (E) or 16 (A, B, C, F, G) hours (N > 3 independent experiments). **p < 0.01, ***p < 0.005 and ****p < 0.0001, 1 or 5 mM APAP *vs.* non-treated cells

Next*,* we evaluated APAP-induced cell death in immortalized neonatal mouse hepatocytes. These cells were exposed to APAP (1 mM and 5 mM) for 16 h as previously described (Mobasher et al. 2013). As shown in Fig. 3A, we observed a decrease in cell viability in a concentration-dependent manner. Moreover, both APAP concentrations had cytotoxic effects reflected by the release of LDH (Fig. 3B) and presence of apoptotic nuclei (Fig. 3C). Furthermore, we used DHE probe to measure reactive oxygen species (ROS) production in these cells during 2 h after APAP stimulation and we found that APAP-induced ROS (Fig. 3D) and, thus, ROS-mediated JNK and P38 MAPK phosphorylation was also observed (Fig. 3E). Next, we measured *Bmp6* mRNA levels and found a significant increase in hepatocytes treated with APAP compared to the controls (Fig. 3F). In addition, BMP6 was detected in the culture media (CM) from cells exposed to APAP (Fig. 3G). Interestingly, a time course experiment revealed that after 16 h, the increased BMP6 gene expression was lower, but the secretion of this BMP was maintained over time (Supplementary Fig. 1), indicating that the cellular expression and secretion profile of BMP6 upon APAP challenge was similar to that found in APAP-treated mice between its hepatic expression and circulating concentration.

### Human hepatocytes express and release BMP6 in the presence of APAP

**Fig. 4:**
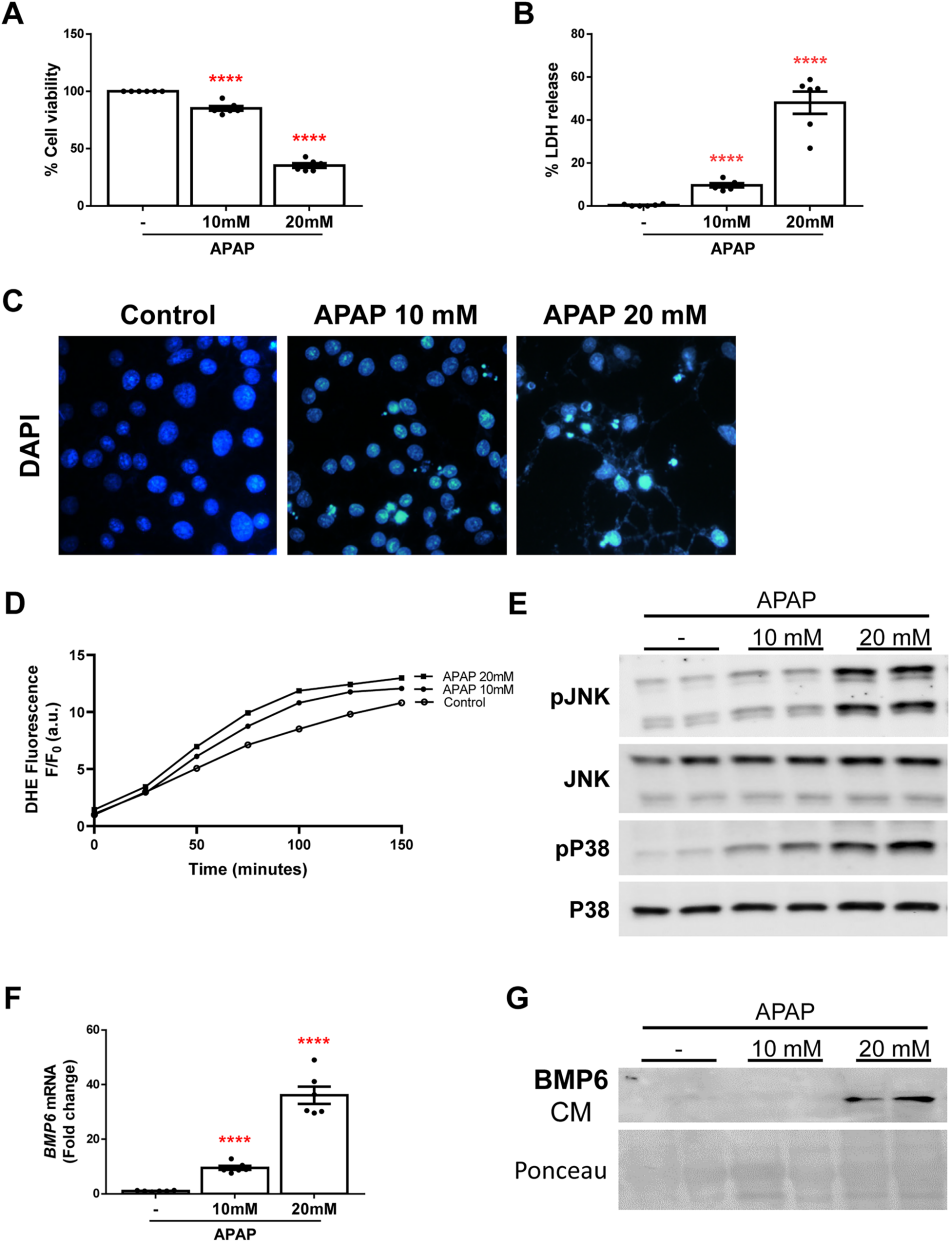
Huh7 hepatocytes release BMP6 after APAP exposure. **A** Cell viability determined by crystal violet staining. Data are expressed as percentage relative to control condition (100%). **B** Cytotoxicity determined by lactate dehydrogenase (LDH) release. Data are expressed as percentage relative to the positive control (100%). **C** Representative 40X images of DAPI staining. **D** ROS production detected with DHE probe represented as DHE fluorescence F/F_0_ (a.u.). **E** Representative blots of the cell lysates with the indicated antibodies. **F** *BMP6* mRNA levels determined by RT-qPCR and normalized to *36B4* gene expression. Data are expressed as fold of change relative to control condition. **G** Representative blot of the cultured media (CM) with BMP6 antibody. Ponceau staining was used as loading control. Experimental conditions: Huh7 treated with 10 mM and 20 mM APAP for 2 (D), 6 (E) or 16 (A, B, C, F, G) hours (*N* > 3 independent experiments). ****p < 0.0001, 10 or 20 mM APAP *vs.* non-treated cells

In order to evaluate if these results could be also observed in human hepatocytes, we evaluated APAP-induced cell death in Huh7 hepatocytes. These cells were treated with 10 mM or 20 mM APAP for 16 h and the hepatotoxic effect was observed by crystal violet staining, LDH release and DAPI staining (Fig. 4A–C). Also, ROS production (Fig. 4D) and phosphorylation of the kinases JNK and P38 (Fig. 4E) after APAP treatment were found. Moreover, *BMP6* mRNA expression increased in a dose-dependent manner (Fig. 4F) and BMP6 was exclusively detected in the CM of APAP-treated Huh7 cells at 20 mM (Fig. 4G). Since similar effect regarding BMP6 secretion upon APAP stimuli was found in mouse hepatocytes and in the human hepatoma cell line Huh7, we used Huh7 cells for further experiments.

### Silencing of BMP6 in hepatocytes does not affect APAP-induced cell death

**Fig. 5:**
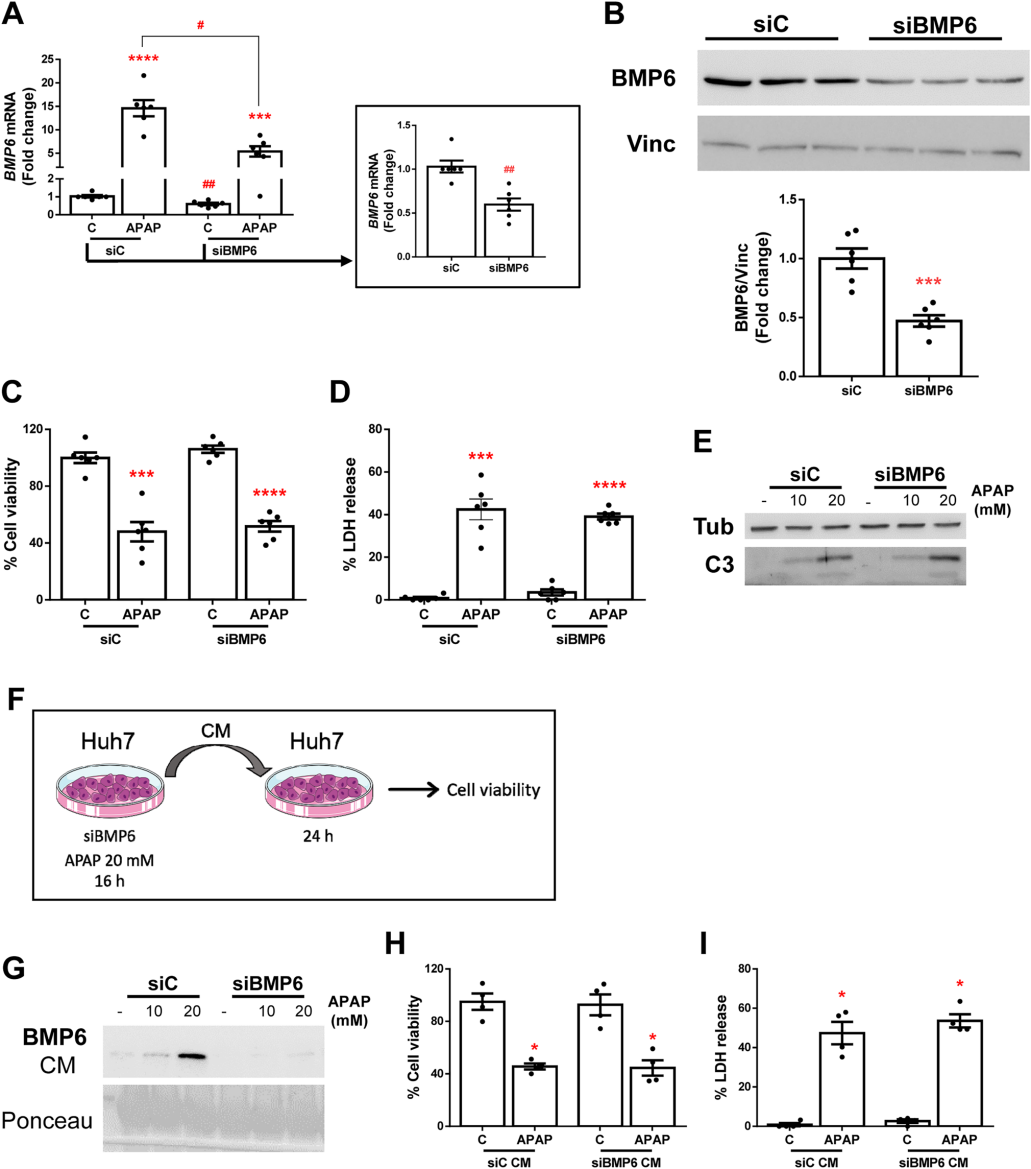
Silencing of BMP6 in Huh7 cells does not affect APAP-induced cell death. Experimental conditions (A–E): siBMP6 and control (siC) Huh7 cells treated with 20 mM APAP for 16 h (*N* > 3 independent experiments). **A** *BMP6* mRNA levels determined by RT-qPCR and normalized to *36B4* gene expression. Data are expressed as fold of change relative to control condition (siC-C). **B** Representative blots of the cell lysates with the indicated antibodies and the corresponding quantification. Data are expressed as fold of change relative to control condition (siC). **C** Cell viability determined by crystal violet staining. Data are expressed as percentage relative to control condition (C, 100%). **D** Cytotoxicity determined by lactate dehydrogenase (LDH) release. Data are expressed as percentage relative to the positive control (100%). **E** Representative blots of the cell lysates with the indicated antibodies. **F** Experimental conditions (F–I): siBMP6 and control (siC) Huh7 cells treated with 20 mM APAP for 16 h. Huh7 cells incubated with siBMP6- or siC-CM for 24 h (*N* > 3 independent experiments). **G** Representative blot of the cultured media (CM) with BMP6 antibody. Ponceau staining was used as loading control. **H**. Cell viability determined by crystal violet staining. Data are expressed as percentage relative to control condition (C, 100%). **I** Cytotoxicity determined by lactate dehydrogenase (LDH) release. Data are expressed as percentage relative to the positive control (100%). *p < 0.05, ***p < 0.005 and ****p < 0.0001, APAP *vs.* C; ^#^*p* < 0.05 and ^##^*p* < 0.01, siBMP6 *vs.* siC

Given the significant increase of BMP6 expression found in hepatocytes upon APAP treatment, as well as BMP6 release to the CM, we sought to explore its biological function. To this end, we conducted a transient BMP6 silencing in Huh7 cells prior APAP exposure. As shown in Fig. 5A and B, mRNA and protein levels of BMP6 were reduced by 50%, approximately, and the induction of its expression by APAP was lower in BMP6-silenced cells (siBMP6) than in non-silenced ones (siControl, siC) (Fig. 5A). However, reduction of BMP6 did not affect cell survival after treatment with APAP (Fig. 5C) and, also, no differences were found between control cells and *BMP6*-silenced hepatocytes in LDH release or in caspase-3 cleavage, a well-known marker of apoptosis (Fig. 5D and E).

Due to the fact that BMP6 is released to the CM by the hepatocytes, we hypothesized on a paracrine effect of this protein. To ascertain this possibility, CM from APAP-induced *BMP6*- silenced or control Huh7 was collected and used to treat fresh Huh7 cells (Fig. 5F). Despite of the partial BMP6 silencing in Huh7 hepatocytes, we did not detect the presence of BMP6 in the CM of these cells after treatment with APAP (20 mM) (Fig. 5G). Nevertheless, no differences in cell viability and toxicity were found in Huh7 hepatocytes regardless of the presence of BMP6 in the CM (Fig. 5H and I).

### BMP6 promotes differentiation of THP1 cells to an anti-inflammatory phenotype (M2)

**Fig. 6:**
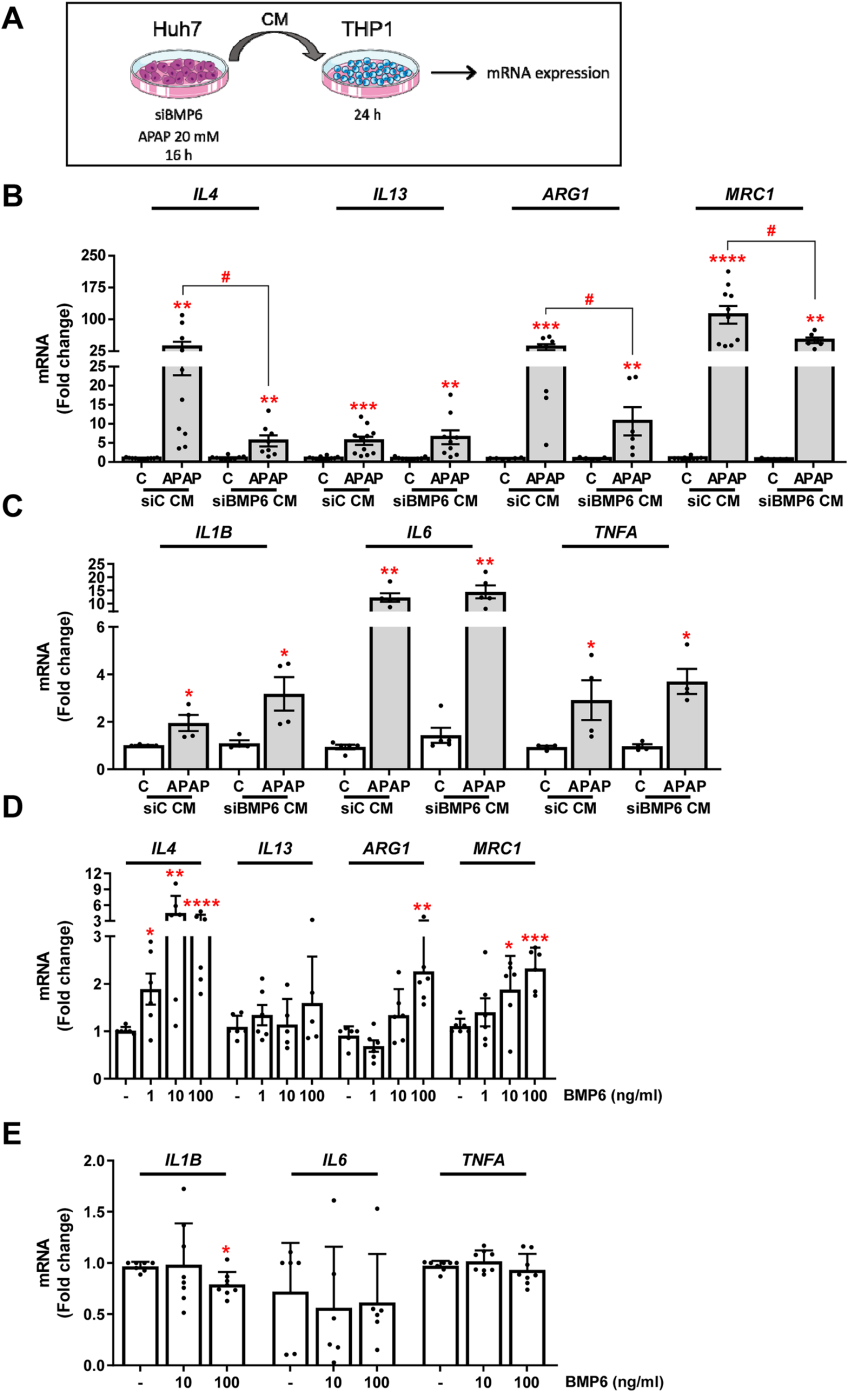
BMP6 promotes THP1 polarization toward an anti-inflammatory phenotype (M2). **A** Experimental conditions: siBMP6 and control (siC) Huh7 hepatocytes treated with 20 mM APAP for 16 h. THP1 cells incubated with siBMP6- or siC-CM for 24 h (N > 3 independent experiments). **B, C, D and E** mRNA levels of *IL4, IL13, ARG1, MRC1, IL1B, IL6* and *TNFA* determined by RT-qPCR and normalized to *36B4* gene expression. Data are expressed as fold of change relative to the control condition (C or -). Experimental conditions: (**D and E**) THP1 treated with BMP6 (1, 10 and 100 ng/ml) for 24 h (*N* > 3 independent experiments performed by duplicate). *p < 0.05, **p < 0.01, ***p < 0.005 and ****p < 0.0001, APAP *vs.* C, or BMP6 *vs.* -; ^#^*p* < 0.05 siBMP6 CM *vs.* siC CM

Since released BMP6 showed no effect on hepatocytes, we studied a possible effect on other cell types present in the liver tissue. For this purpose, we exposed the human monocyte cell line THP1 for 24 h to the CM collected from Huh7 with or without *BMP6* silencing treated with 20 mM APAP for 16 h (Fig. 6A). As shown in Fig. 6B and C, a significant increase of both pro- and anti-inflammatory markers was observed after exposure of THP1 cells to the CM from APAP-treated Huh7 hepatocytes. However, the CM from *BMP6*-silenced cells significantly attenuated the increase in the expression of some anti-inflammatory cytokines, such as interleukin (IL) 4, arginase 1 and MRC1, but not IL13. Moreover, no significant changes of pro-inflammatory cytokines, such as IL1β, IL6 and TNFα, were found (Fig. 6B and C).

In order to clarify whether the modulation of the inflammatory profile of THP1 monocytes was associated with the presence of BMP6 in the CM, we directly treated THP1 cells with BMP6 (1, 10 or 100 ng/ml) for 24 h. As shown in Fig. 6D, expression levels of the anti-inflammatory cytokine *IL4* and the anti-inflammatory markers *ARG1* and *MRC1* were significantly upregulated after the treatment with BMP6, while the expression of *IL13* was unaltered. Notably, the expression of the pro-inflammatory markers *IL1B, IL6* and *TNFA* was unchanged (Fig. 6E). These results indicate that BMP6 released from the hepatocytes could have an impact on macrophages by promoting an anti-inflammatory phenotype (M2).

## Discussion

In this study, we provide convincing data showing that BMP6 might be a novel biomarker and a potential pharmacological target in DILI due to APAP overdose. On the one hand, expression profile of hepatic and circulating BMP6 is upregulated during APAP-induced acute liver injury and, on the other hand, this protein contributes to the molecular processes involved in APAP hepatotoxicity.

First, we found increased hepatic expression of BMP6 in an animal model of APAP-induced ALF, similar to previous studies which reported increased expression of other BMP family members (BMP2 and BMP4) in animal models of acute liver injury where BMP signaling seems to play an important role in the repairing process by promoting the proliferation of hepatic cells (Nakatsuka et al. 2007; Oumi et al. 2012). In fact, BMP4 signaling inhibition by knocking-down ALK3 receptor reduced the repair liver capacity after damage and lowered the expression of genes related to cell proliferation (Oumi et al. 2012). This study, at a glance, reported contrary results that those published by Stavropoulos et al. (Stavropoulos et al. 2022), indicating that inhibition of TGFβ superfamily signaling accelerated liver tissue recovery after the induction of worse damage. These contradictory results might be explained since in the latter study, the inhibition of TGFβ superfamily signaling was achieved by SMAD7 overexpression, which blocks both TGFβ and BMP signals, or by treatment with LY364947, a selective TGFβ receptor, which only blocks TGFβ signaling. Thus, both approaches inhibited TGFβ signaling and the first mentioned study (Oumi et al. 2012) also explored the inhibition of BMP pathway. On that basis, we can conclude that although a coordinated and cooperative activation of BMP and TGFβ pathways controls APAP-induced liver damage, the selective inhibition of each pathway differentially impacts on acute liver injury.

Recently, BMP6 has been widely studied for its key role in regulating iron homeostasis in the liver since it modulates hepcidin expression in situations of iron overload (Canali et al. 2017). Indeed, hepatic BMP6 production was classically attributed to non-parenchymal cells (NPCs), such as hepatic stellate cells, Kupffer cells and liver sinusoidal epithelial cells (Knittel et al. 1997; Enns et al. 2013). However, hepatocyte-derived BMP6 production has been also demonstrated. In this regard, murine hepatocytes produced BMP6 in an in vitro model of cellular lipid accumulation and hepatocytes were identified as a cellular source of hepatic BMP6 in livers of patients with non-alcoholic fatty liver disease (Arndt et al. 2015). Moreover, chronic dietary iron changes were reported to modulate liver BMP6 expression in all cell types, including hepatocytes (Rausa et al. 2015). In fact, BMP6 expression induced by iron has been found in mouse primary hepatocytes, human hepatocytes and human hepatocellular carcinoma (Kim et al. 2021). Other study has confirmed by RT-PCR and immunohistochemistry that hepatocytes produce BMP6 in IL6-treated mice (Radhakrishnan et al. 2020). Until now, only one study has evaluated hepatic BMP6 expression in a model of APAP-induced DILI. Contrary to our results, van Swelm et al*.* reported reduced BMP6 expression in the liver of mice challenged with APAP (Swelm et al. 2012). This discrepancy could be explained because those authors determined hepatic BMP6 expression in mice sacrificed 24 h after APAP, whereas we observed the maximal hepatic mRNA expression of BMP6 after 6 h of APAP administration, being markedly reduced after 24 h.

One of the most striking findings of this study was that serum levels of BMP6 positively correlated with the hepatic expression of this BMP in APAP-treated mice and, importantly, with serum ALT values, a well-known marker of liver damage. In fact, circulating BMP6 in patients exposed to an APAP overdose showed a positive and significant correlation with serum ALT; since this is a proof-of-concept in a small sample, the analysis of circulating BMP6 in larger and well-characterized cohorts might shed light into the possible value of this BMP as a suitable biomarker for monitoring APAP overdose. The fulminant progression featuring ALF makes decisive to establish an early diagnosis and an accurate prognosis in order to choose the best therapeutic approach. In this regard, the search of new serological biomarkers is of great importance. Nowadays, several biomarkers, such as GLDH, microRNA-122 or K18, have been proposed as prognosis markers of early liver injury in APAP-induced DILI. Currently, evaluation of APAP-cysteine adducts (APAP-CYS) is the most promising biomarker for DILI diagnosis.

On the other hand, this study provides new insights into the role of BMP6 on the inflammatory response activated upon liver damage by APAP, more specifically on the crosstalk between hepatocytes and macrophages. An increase in the expression of BMP6 was observed in mouse hepatocytes and Huh7 cells after APAP treatment, as well as the release of this protein into the CM. Although transient silencing of *BMP6* in hepatocytes did not alter the APAP cytotoxicity, the stimulation of THP1 monocytes with the CM derived from APAP-treated hepatocytes induced their differentiation toward both M1 and M2 phenotypes, whereas the CM derived from hepatocytes with reduced BMP6 expression levels attenuated the enhancement in M2 polarization. These results suggest that during hepatocyte necrosis, DAMPs release induces a potent inflammatory response and BMP6 is released to maintain a balanced M1/M2 polarization pattern to counteract the inflammatory response. In fact, the absence of BMP6 in the CM of APAP-treated hepatocytes impaired this M2 response likely promoting the inflammatory loop upon APAP challenge. The relevance of BMP6 was reinforced by the increase of M2 response in TPH1 monocytes directly treated with BMP6. As reported (Chung et al. 2012; Ramachandran and Jaeschke 2018), massive hepatocyte necrosis due to APAP toxicity leads to the release of DAMPs that induce an inflammatory response by activating macrophages. As mentioned above, BMP6, directly or through the APAP-CM, induced the expression of the anti-inflammatory markers *IL4*, *ARG1* and *MRC1* in human THP1 cells without altering the expression of pro-inflammatory cytokines. Likewise, Lee et al*.* found that BMP6 also promoted the expression of IL10, another cytokine that suppresses THP1 inflammation (Lee et al. 2013). These results, observed using human monocytes, are in direct contrast to the previously reported effects of BMP6 on murine macrophages in which BMP6 induced the expression of the pro-inflammatory cytokines TNFα and IL1β (Kwon et al. 2009; Lee et al. 2011). These contradictory results might reside in the different cell types used that need different doses or times. Thus, further investigations are needed in order to elucidate the role of BMP6 in the inflammatory response derived from APAP damage.

In conclusion, we provide evidence that BMP6 might be a new biomarker and a potential pharmacological target for APAP-induced acute liver injury. Moreover, our results suggest that BMP6 might play a role driving the inflammatory crosstalk between hepatocytes and macrophages during APAP hepatotoxicity.

## Supplementary Information

Below is the link to the electronic supplementary material.

Supplementary file1 (DOCX 15 KB)

Supplementary file2 Figure 1. Time-course experiments in immortalized mouse hepatocytes checking BMP6 expression and secretion at different time points. A. *Bmp6* mRNA levels determined by RT-qPCR and normalized to *36b4* gene expression. B. Representative blot of the cultured media (CM) with BMP6 antibody. Ponceau staining was used as loading control. Experimental conditions: immortalized mouse hepatocytes treated with 1 mM or 5 mM APAP for 6, 16, 24 and 36 hours (*N*>3 independent experiments). **p<0.01 and ***p<0.005, 1 or 5 mM APAP vs. C. (TIF 1496 KB)

## Data Availability

Authors declared that all and the other data supporting the findings of this study are available within the paper. The raw data that support the findings of this study are available from the corresponding author upon reasonable request.

## References

[CR1] Andrade RJ, Chalasani N, Björnsson ES, Suzuki A, Kullak-Ublick GA, Watkins PB et al (2019) Drug-induced liver injury. Nat Rev Dis Primers 5(1):58 31439850 10.1038/s41572-019-0105-0

[CR2] Andriopoulos B, Corradini E, Xia Y, Faasse SA, Chen S, Grgurevic L et al (2009) BMP6 is a key endogenous regulator of hepcidin expression and iron metabolism. Nat Genet 41(4):482–487 19252486 10.1038/ng.335 PMC2810136

[CR3] Arndt S, Wacker E, Dorn C, Koch A, Saugspier M, Thasler WE et al (2015) Enhanced expression of BMP6 inhibits hepatic fibrosis in non-alcoholic fatty liver disease. Gut 64(6):973–981 25011936 10.1136/gutjnl-2014-306968

[CR4] Bernal W, Wendon J (2013) Acute liver failure. N Engl J Med 369(26):2525–2534 24369077 10.1056/NEJMra1208937

[CR5] Bunchorntavakul C, Reddy KR (2018) Acetaminophen (APAP or N-Acetyl-p-Aminophenol) and acute liver failure. Clin Liver Dis 22(2):325–346 29605069 10.1016/j.cld.2018.01.007

[CR6] Canali S, Wang CY, Zumbrennen-Bullough KB, Bayer A, Babitt JL (2017) Bone morphogenetic protein 2 controls iron homeostasis in mice independent of Bmp6. Am J Hematol 92(11):1204–1213 28815688 10.1002/ajh.24888 PMC5986189

[CR7] Cardoso FS, Marcelino P, Bagulho L, Karvellas CJ (2017) Acute liver failure: an up-to-date approach. J Crit Care 39:25–30 28131021 10.1016/j.jcrc.2017.01.003

[CR8] Carreira AC, Alves GG, Zambuzzi WF, Sogayar MC, Granjeiro JM (2014) Bone morphogenetic proteins: structure, biological function and therapeutic applications. Arch Biochem Biophys 561:64–73 25043976 10.1016/j.abb.2014.07.011

[CR9] Chung RT, Stravitz RT, Fontana RJ, Schiodt FV, Mehal WZ, Reddy KR et al (2012) Pathogenesis of liver injury in acute liver failure. Gastroenterology 143(3):e1–e7 22796239 10.1053/j.gastro.2012.07.011 PMC3641754

[CR10] Daher R, Kannengiesser C, Houamel D, Lefebvre T, Bardou-Jacquet E, Ducrot N et al (2016) Heterozygous mutations in BMP6 pro-peptide lead to inappropriate hepcidin synthesis and moderate iron overload in humans. Gastroenterology 150(3):672–83.e4 26582087 10.1053/j.gastro.2015.10.049

[CR11] Du K, Ramachandran A, Jaeschke H (2016) Oxidative stress during acetaminophen hepatotoxicity: Sources, pathophysiological role and therapeutic potential. Redox Biol 10:148–156 27744120 10.1016/j.redox.2016.10.001 PMC5065645

[CR12] Enns CA, Ahmed R, Wang J, Ueno A, Worthen C, Tsukamoto H et al (2013) Increased iron loading induces Bmp6 expression in the non-parenchymal cells of the liver independent of the BMP-signaling pathway. PLoS ONE 8(4):e60534 23565256 10.1371/journal.pone.0060534 PMC3615098

[CR14] Heard KJ, Green JL, James LP, Judge BS, Zolot L, Rhyee S et al (2011) Acetaminophen-cysteine adducts during therapeutic dosing and following overdose. BMC Gastroenterol 11:20 21401949 10.1186/1471-230X-11-20 PMC3066114

[CR15] Kautz L, Besson-Fournier C, Meynard D, Latour C, Roth MP, Coppin H (2011) Iron overload induces BMP6 expression in the liver but not in the duodenum. Haematologica 96(2):199–203 20952515 10.3324/haematol.2010.031963 PMC3031686

[CR16] Kim HY, Lee JM, Lee YS, Li S, Lee SJ, Bae SC et al (2021) Runx3 regulates iron metabolism via modulation of BMP signalling. Cell Prolif 54(12):e13138 34611951 10.1111/cpr.13138 PMC8666273

[CR17] Knittel T, Fellmer P, Müller L, Ramadori G (1997) Bone morphogenetic protein-6 is expressed in nonparenchymal liver cells and upregulated by transforming growth factor-beta 1. Exp Cell Res 232(2):263–269 9168801 10.1006/excr.1997.3504

[CR18] Kwon SJ, Lee GT, Lee JH, Kim WJ, Kim IY (2009) Bone morphogenetic protein-6 induces the expression of inducible nitric oxide synthase in macrophages. Immunology 128(1 Suppl):e758–e765 19740337 10.1111/j.1365-2567.2009.03079.x PMC2753926

[CR19] Lee WM, Squires RH, Nyberg SL, Doo E, Hoofnagle JH (2008) Acute liver failure: summary of a workshop. Hepatology 47(4):1401–1415 18318440 10.1002/hep.22177 PMC3381946

[CR20] Lee GT, Jung YS, Lee JH, Kim WJ, Kim IY (2011) Bone morphogenetic protein 6-induced interleukin-1β expression in macrophages requires PU.1/Smad1 interaction. Mol Immunol. 48(12–13):1540–7 21571370 10.1016/j.molimm.2011.04.019

[CR21] Lee JH, Lee GT, Woo SH, Ha YS, Kwon SJ, Kim WJ et al (2013) BMP-6 in renal cell carcinoma promotes tumor proliferation through IL-10-dependent M2 polarization of tumor-associated macrophages. Cancer Res 73(12):3604–3614 23633487 10.1158/0008-5472.CAN-12-4563

[CR22] Llewellyn HP, Vaidya VS, Wang Z, Peng Q, Hyde C, Potter D et al (2021) Evaluating the sensitivity and specificity of promising circulating biomarkers to diagnose liver injury in humans. Toxicol Sci 181(1):23–34 33483742 10.1093/toxsci/kfab003

[CR23] McGill MR, Jaeschke H (2018) Biomarkers of drug-induced liver injury: progress and utility in research, medicine, and regulation. Expert Rev Mol Diagn 18(9):797–807 30080986 10.1080/14737159.2018.1508998 PMC6288799

[CR24] Meynard D, Kautz L, Darnaud V, Canonne-Hergaux F, Coppin H, Roth MP (2009) Lack of the bone morphogenetic protein BMP6 induces massive iron overload. Nat Genet 41(4):478–481 19252488 10.1038/ng.320

[CR25] Mobasher MA, González-Rodriguez A, Santamaría B, Ramos S, Martín M, Goya L et al (2013) Protein tyrosine phosphatase 1B modulates GSK3β/Nrf2 and IGFIR signaling pathways in acetaminophen-induced hepatotoxicity. Cell Death Dis 4(5):e626 23661004 10.1038/cddis.2013.150 PMC3674359

[CR26] Nakatsuka R, Taniguchi M, Hirata M, Shiota G, Sato K (2007) Transient expression of bone morphogenic protein-2 in acute liver injury by carbon tetrachloride. J Biochem 141(1):113–119 17158861 10.1093/jb/mvm012

[CR27] Oumi N, Taniguchi KA, Kanai AM, Yasunaga M, Nakanishi T, Sato K (2012) A crucial role of bone morphogenetic protein signaling in the wound healing response in acute liver injury induced by carbon tetrachloride. Int J Hepatol 2012:476820 22701178 10.1155/2012/476820 PMC3372049

[CR28] Pardo V, González-Rodríguez Á, Muntané J, Kozma SC, Valverde Á (2015) Role of hepatocyte S6K1 in palmitic acid-induced endoplasmic reticulum stress, lipotoxicity, insulin resistance and in oleic acid-induced protection. Food Chem Toxicol 80:298–309 25846498 10.1016/j.fct.2015.03.029

[CR29] Rada P, Pardo V, Mobasher MA, García-Martínez I, Ruiz L, González-Rodríguez Á et al (2018) SIRT1 controls acetaminophen hepatotoxicity by modulating inflammation and oxidative stress. Antioxid Redox Signal 28(13):1187–1208 29084443 10.1089/ars.2017.7373 PMC9545809

[CR30] Radhakrishnan K, Kim YH, Jung YS, Kim J, Kim DK, Cho SJ, et al. Orphan Nuclear Receptor ERRγ Is a Novel Transcriptional Regulator of IL-6 Mediated Hepatic BMP6 Gene Expression in Mice. Int J Mol Sci. 2020;21(19). 10.3390/ijms21197148 PMC7582774 32998264

[CR31] Ramachandran A, Jaeschke H (2018) Acetaminophen toxicity: novel insights into mechanisms and future perspectives. Gene Expr 18(1):19–30 29054140 10.3727/105221617X15084371374138 PMC5885144

[CR32] Rausa M, Pagani A, Nai A, Campanella A, Gilberti ME, Apostoli P et al (2015) Bmp6 expression in murine liver non parenchymal cells: a mechanism to control their high iron exporter activity and protect hepatocytes from iron overload? PLoS ONE 10(4):e0122696 25860887 10.1371/journal.pone.0122696 PMC4393274

[CR33] Reid AB, Kurten RC, McCullough SS, Brock RW, Hinson JA (2005) Mechanisms of acetaminophen-induced hepatotoxicity: role of oxidative stress and mitochondrial permeability transition in freshly isolated mouse hepatocytes. J Pharmacol Exp Ther 312(2):509–516 15466245 10.1124/jpet.104.075945

[CR34] Silvestri L, Nai A, Dulja A, Pagani A (2019) Hepcidin and the BMP-SMAD pathway: an unexpected liaison. Vitam Horm 110:71–99 30798817 10.1016/bs.vh.2019.01.004

[CR35] Simonse E, Valk-Swinkels CG, van’t Veer NE, Ermens AA, Veldkamp EJ (2013) Iron autointoxication in a 16-year-old girl: a protective role for hepcidin? Ann Clin Biochem. 50(Pt 1):76–9 23108765 10.1258/acb.2012.012038

[CR36] Spivak I, Arora J, Meinzer C, Durkalski-Mauldin V, Lee WM, Trautwein C et al (2019) Low serum hepcidin is associated with reduced short-term survival in adults with acute liver failure. Hepatology 69(5):2136–2149 30582749 10.1002/hep.30486

[CR37] Stavropoulos A, Divolis G, Manioudaki M, Gavriil A, Kloukina I, Perrea DN et al (2022) Coordinated activation of TGF-β and BMP pathways promotes autophagy and limits liver injury after acetaminophen intoxication. Sci Signal. 15(740):eabn4395 35763560 10.1126/scisignal.abn4395

[CR38] Umbaugh DS, Jaeschke H (2021) Biomarkers of drug-induced liver injury: a mechanistic perspective through acetaminophen hepatotoxicity. Expert Rev Gastroenterol Hepatol 15(4):363–375 33242385 10.1080/17474124.2021.1857238 PMC8026489

[CR39] van Swelm RP, Laarakkers CM, Blous L, Peters JG, Blaney Davidson EN, van der Kraan PM et al (2012) Acute acetaminophen intoxication leads to hepatic iron loading by decreased hepcidin synthesis. Toxicol Sci 129(1):225–233 22610607 10.1093/toxsci/kfs176

[CR40] Vliegenthart AD, Antoine DJ, Dear JW (2015) Target biomarker profile for the clinical management of paracetamol overdose. Br J Clin Pharmacol 80(3):351–362 26076366 10.1111/bcp.12699 PMC4574821

[CR41] Whitehouse T, Wendon J (2013) Acute liver failure. Best Pract Res Clin Gastroenterol 27(5):757–769 24160932 10.1016/j.bpg.2013.08.010

[CR42] Wozney JM, Rosen V, Celeste AJ, Mitsock LM, Whitters MJ, Kriz RW et al (1988) Novel regulators of bone formation: molecular clones and activities. Science 242(4885):1528–1534 3201241 10.1126/science.3201241

[CR43] Xiao YT, Xiang LX, Shao JZ (2007) Bone morphogenetic protein. Biochem Biophys Res Commun 362(3):550–553 17719560 10.1016/j.bbrc.2007.08.045

[CR44] Yan M, Huo Y, Yin S, Hu H (2018) Mechanisms of acetaminophen-induced liver injury and its implications for therapeutic interventions. Redox Biol 17:274–283 29753208 10.1016/j.redox.2018.04.019 PMC6006912

