# Supplementary material for: BMP6 participates in the molecular mechanisms involved in APAP hepatotoxicity
Source: Arch Toxicol. 2025 Jan 19;99(3):1187–202. doi: 10.1007/s00204-024-03954-5 (PMC11821676; doi:10.1007/s00204-024-03954-5)
Supplement: Supplementary file 1 — Supplementary file1 (DOCX 15 KB) [file 204_2024_3954_MOESM1_ESM.docx]

**Supplementary Table 1. Primer sequences for RT-qPCR.**

| **Gene** | **Forward (5’-3’)** | **Reverse (5’-3’)** |
| --- | --- | --- |
| *m-Hmox* | CACAGATGGCGTCACTTCGTC | GTGAGGACCCACTGGAGGAG |
| *m-Bmp6* | TCCCCACATCAACGACACCA | TCCCCACCACACAGTCCTTG |
| *m-36b4* | AGATGCAGCAGATCCGCAT | GTTCTTGCCATCAGCACC |
| *h-BMP6* | GGAAGCATGAGCTGTATGTGAGTTT | AGTAATTGGCAGCATAGCATAGCCCTTG |
| *h-IL-4* | CCGTAACAGACATCTTTGCTGCC | GAGTGTCCTTCTCATGGTGGCT |
| *h-IL-13* | ACGGTCATTGCTCTCACTTGCC | CTGTCAGGTTGATGCTCCATACC |
| *h-ARG1* | TCATCTGGGTGGATGCTCACAC | GAGAATCCTGGCACATCGGGAA |
| *h-MRC1* | AGCCAACACCAGCTCCTCAAGA | CAAAACGCTCGCGCATGTCCA |
| *h-IL-1β* | TGAGCACCTTCTTTCCCTTCA | ATCGTGCACATAAGCCTCGTT |
| *h-IL-6* | CCTGACCCAACCACAAATGC | CCTTAAAGCTGCGCAGAATGA |
| *h-TNFα* | TCGAACCCCGAGTGACAAG | TTGGCCAGGAGGGCATT |
| *h-36B4* | CAGGCGTCCTCGTGGAAGTGAC | CCAGGTCGCCCTGTCTTCCCT |
