# Supplementary figures and images for: BMP6 participates in the molecular mechanisms involved in APAP hepatotoxicity
Source: Arch Toxicol. 2025 Jan 19;99(3):1187–202. doi: 10.1007/s00204-024-03954-5 (PMC11821676; doi:10.1007/s00204-024-03954-5)

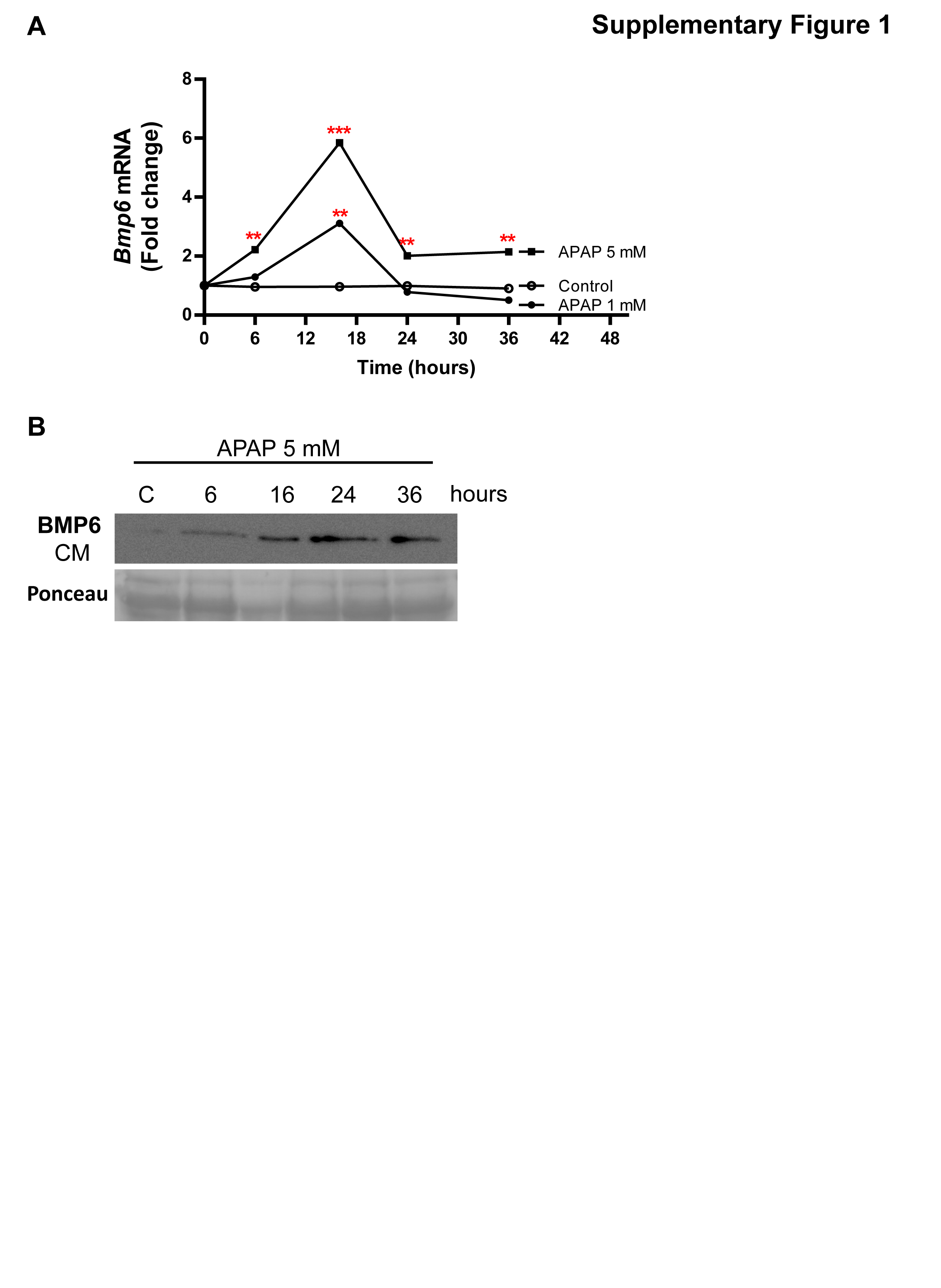

Supplement: Supplementary file 2 — Supplementary file2 Figure 1. Time-course experiments in immortalized mouse hepatocytes checking BMP6 expression and secretion at different time points. A. Bmp6 mRNA levels determined by RT-qPCR and normalized to 36b4 gene expression. B. Representative blot of the cultured media (CM) with BMP6 antibody. Ponceau staining was used as loading control. Experimental conditions: immortalized mouse hepatocytes treated with 1 mM or 5 mM APAP for 6, 16, 24 and 36 hours (N>3 independent experiments). **p<0.01 and ***p<0.005, 1 or 5 mM APAP vs. C. (TIF 1496 KB) [file 204_2024_3954_MOESM2_ESM.tif]
